# Peroxisomal abnormalities in the immortalized human hepatocyte (IHH) cell line

**DOI:** 10.1007/s00418-016-1532-6

**Published:** 2016-12-24

**Authors:** Femke C. C. Klouwer, Janet Koster, Sacha Ferdinandusse, Hans R. Waterham

**Affiliations:** 1 Laboratory Genetic Metabolic Diseases, Academic Medical Center, University of Amsterdam, Meibergdreef 9, 1105 AZ Amsterdam, The Netherlands; 2 Department of Pediatric Neurology, Emma Children’s Hospital, Academic Medical Center, University of Amsterdam, Amsterdam, The Netherlands

**Keywords:** Immortalized human hepatocyte cell line, Peroxisomal abnormalities, Plasmalogen deficiency, Lipid metabolism

## Abstract

The immortalized human hepatocyte (IHH) cell line is increasingly used for studies related to liver metabolism, including hepatic glucose, lipid, lipoprotein and triglyceride metabolism, and the effect of therapeutic interventions. To determine whether the IHH cell line is a good model to investigate hepatic peroxisomal metabolism, we measured several peroxisomal parameters in IHH cells and, for comparison, HepG2 cells and primary skin fibroblasts. This revealed a marked plasmalogen deficiency and a deficient fatty acid α-oxidation in the IHH cells, due to a defect of PEX7, a cytosolic receptor protein required for peroxisomal import of a subset of peroxisomal proteins. These abnormalities have consequences for the lipid homeostasis of these cells and thus should be taken into account for the interpretation of data previously generated by using this cell line and when considering using this cell line for future research.

## Introduction

Immortalized human hepatocyte cell lines are frequently used as in vitro models to study liver metabolism in health and disease, pharmacokinetics and the efficacy of therapeutic interventions. Different immortalization strategies have been described to generate hepatocyte cell lines from liver samples (Ramboer et al. 2014). One hepatocyte cell line used in several published studies is the immortalized human hepatocyte (IHH) cell line, which was established by stable transfection of human hepatocytes with simian virus 40 large T antigen (SV40 Tag) (Schippers et al. 1997). Among others, this IHH cell line has been used to study hepatic glucose, lipid, lipoprotein and triglyceride metabolism and the effect of therapeutic interventions (Perttilä et al. 2012; Samanez et al. 2012; Sukowati et al. 2012) and recently is gaining increasing research interest (Jansen et al. 2016; Nelson et al. 2016; Weider et al. 2016).

Here we studied whether the IHH cell line is a good cell model to study hepatic peroxisomal metabolism. Peroxisomes are organelles involved in a number of essential metabolic pathways involving lipid homeostasis, including the β-oxidation of a variety of fatty acids and α-oxidation of phytanic acid, and the synthesis of plasmalogens and bile acids (Wanders and Waterham 2006). The peroxisomal enzymes involved in these pathways are directed to the peroxisomal matrix by virtue of one of two defined peroxisomal targeting signals, PTS1 or PTS2, which are recognized by the cytosolic receptor proteins PEX5 and PEX7, respectively. The PEX5 receptor is involved in the import of the majority of peroxisomal matrix proteins, and consequently, a defect of PEX5 results in a generalized protein import defect affecting multiple metabolic pathways and leading to a Zellweger spectrum disorder (Dodt et al. 1995). The PEX7 receptor is involved in the import of only a subset of matrix proteins, including alkyl-dihydroxyacetone phosphate (DHAP) synthase, peroxisomal 3-ketoacyl-CoA thiolase and phytanoyl-CoA hydroxylase. Accordingly, a defect of PEX7 only affects the import of these proteins and the metabolic pathways in which these proteins participate, leading to a different disease entity called rhizomelic chondrodysplasia punctata (RCDP) type 1 (Braverman et al. 1997).

We found that the IHH cell line resembled cells from RCDP patients and has a marked plasmalogen deficiency and a deficient fatty acid α-oxidation due to a complete absence of PEX7. Our findings have important implications for the future use of this cell line and interpretation of previously reported results.

## Materials and methods

### Cell culturing

HepG2 cells (obtained from ATCC), IHH-A5 cells (Schippers et al. 1997; kindly provided by Dr. Oosterveer from the University Medical Center Groningen, The Netherlands) and patient primary skin fibroblasts were cultured at 37 °C under an atmosphere of 5% CO_2_. Patient skin fibroblasts were obtained according to standard procedures, and identifiable clinical and personal data from the patients were not available for this study. HepG2 cells and patient skin fibroblasts were cultured in Dulbecco’s modified Eagle’s medium (DMEM) supplemented with l-glutamine (BioWhittaker), 10% fetal bovine serum (Life Technologies), 25 mM HEPES buffer (BioWhittaker), 100 U/mL penicillin (Life Technologies), 100 μg/mL streptomycin (Life Technologies) and 250 ng/mL Fungizone (Life Technologies). IHH cells were cultured in Williams E medium (Life Technologies), supplemented with 10% fetal bovine serum (Life Technologies), 100 U/mL penicillin (Life Technologies), 100 μg/mL streptomycin (Life Technologies), 250 ng/ml Fungizone (Life Technologies), 20 mU/mL insulin as part (Novo Nordisk) and 50 nM/L dexamethasone (Sigma-Aldrich D4902). IHH cells were cultured in 0.1% gelatin-coated (from porcine skin, Sigma-Aldrich G1890) culture flasks.

### Immunofluorescence assays

Immunofluorescence microscope analysis was performed on IHH cells and HepG2 cells.

The cells were cultured on glass slides to a confluency of approximately 50%, and IHH cells were cultured on 0.1% gelatin-coated glass slides. Cells were stained as described (van Grunsven et al. 1999). The peroxisomal matrix protein catalase was labeled with a mouse monoclonal antibody against catalase (in-house generation, 1:4 diluted), biotinylated α-mouse antibody (Dako E433, 1:200 diluted) and streptavidine-FITC (Dako F422, 1:200). The peroxisomal matrix protein 3-ketoacyl-CoA thiolase was labeled with a rabbit polyclonal antibody against thiolase (Atlas antibodies HPA007244, 1:200 diluted), biotinylated α-rabbit antibody (Dako E432, 1:500 diluted) and streptavidine-FITC (Dako F422, 1:200 diluted). Images were taken with a Zeiss Axio Observer A1 fluorescence microscope.

### Western blot analysis

Immunoblot analysis was performed with homogenates of IHH cells, HepG2 cells and skin fibroblasts. Primary skin fibroblasts homozygous for *PEX7* c.694C>T (p.R232X) were used as a PEX7-deficient control. For homogenization, cell pellets were suspended in 500 μL of lysis buffer [PBS, 0.25% Triton X-100 (BioRad), protease inhibitor cocktail tablet (Roche, Mannheim, Germany)] and sonicated twice (8 W, 40 J) on ice water. Proteins were separated by SDS-polyacrylamide gel electrophoresis and subsequently transferred onto a nitrocellulose membrane using semidry blotting. A rabbit polyclonal antibody against thiolase (Atlas antibodies HPA007244) and a rabbit polyclonal antibody against alkyl-DHAP synthase were used [in-house generation (Biermann et al. 1999)] at a 1:2000 solution. A rabbit polyclonal antibody against the c terminus of PEX7 (kindly provided by prof. Y. Fujiki, Kyushu University, Fukuoka, Japan) was used at a 1:1000 solution. For visualization, we used the secondary antibodies IRDye 800 CW goat anti-rabbit (1:10.000) with the Odyssey Infrared Imaging System (LI-COR Biosciences).

### Biochemical and enzyme activity assays

The α-oxidation rate of phytanic acid, and the β-oxidation rates of cerotic acid (C26:0) and pristanic acid were measured in IHH, HepG2 and skin fibroblasts using radioactive labeled substrate as described (Wanders and Van Roermund 1993; Wanders et al. 1995). Plasmalogen levels were measured in pellets of IHH and HepG2 cells and in skin fibroblasts as described (Dacremont and Vincent 1995).

### Mutation analysis

Genomic DNA was isolated using the NucleoSpin Tissue Genomic DNA purification kit (Macherey–Nagel). All exons plus flanking intronic sequences of the *PEX*7 gene were amplified using specific primers for *PEX7* tagged with a -21M13 (5′-TGTAAAACGACGGCCAGT-3′) sequence or M13rev (5′-CAGGAAACAGCTATGACC-3′) sequence. Sequence analysis was performed with the Big DyeTM Terminator v.3.1 Cycle Sequencing Kit (Applied Biosystems) on an ABI 3730 sequencer (Applied Biosystems) using -21M13 or M13rev primers.

### Complementation assay

We performed genetic complementation of IHH cells by transfecting the cells with PEX7 and PEX5L cDNA as described (Ebberink et al. 2011). PTS2-mediated peroxisomal protein import was assessed by co-transfection of the cells with a plasmid encoding PTS2-GFP. Transfection was performed with Lipofectamine 2000 transfection reagent (Thermo Fisher). The subcellular localization of the PTS2-GFP was determined three days after transfection by using the Zeiss Axio Observer A1 fluorescence microscope.

## Results and discussion

**Fig. 1:**
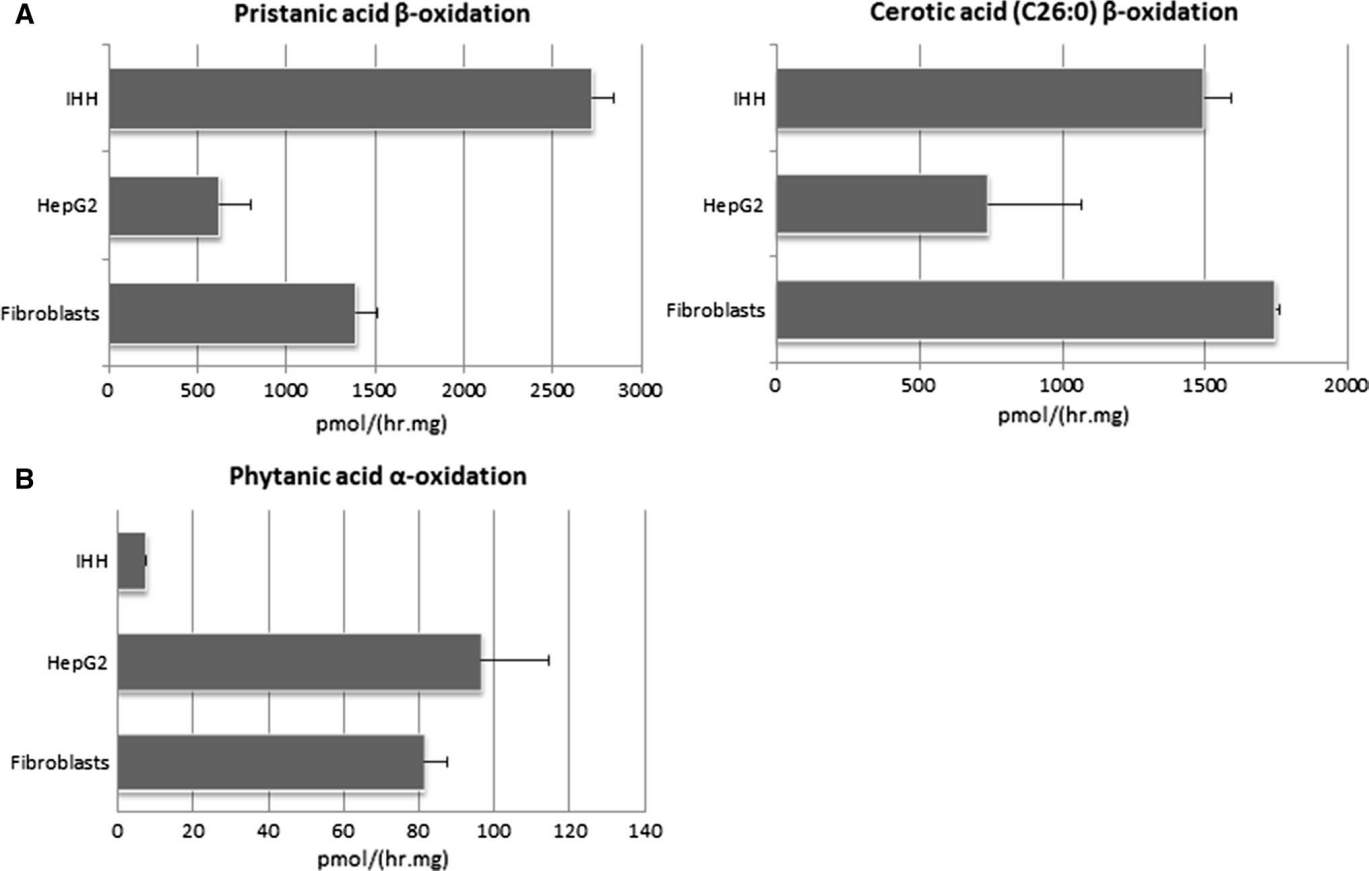
**a** Pristanic acid and cerotic acid (C26:0) β-oxidation [in pmol/(hr.mg)] and **b** phytanic acid α-oxidation [in pmol/(hr.mg)] in IHH cells, HepG2 cells and control human skin fibroblasts, measured using radioactive labeled substrate

**Fig. 2:**
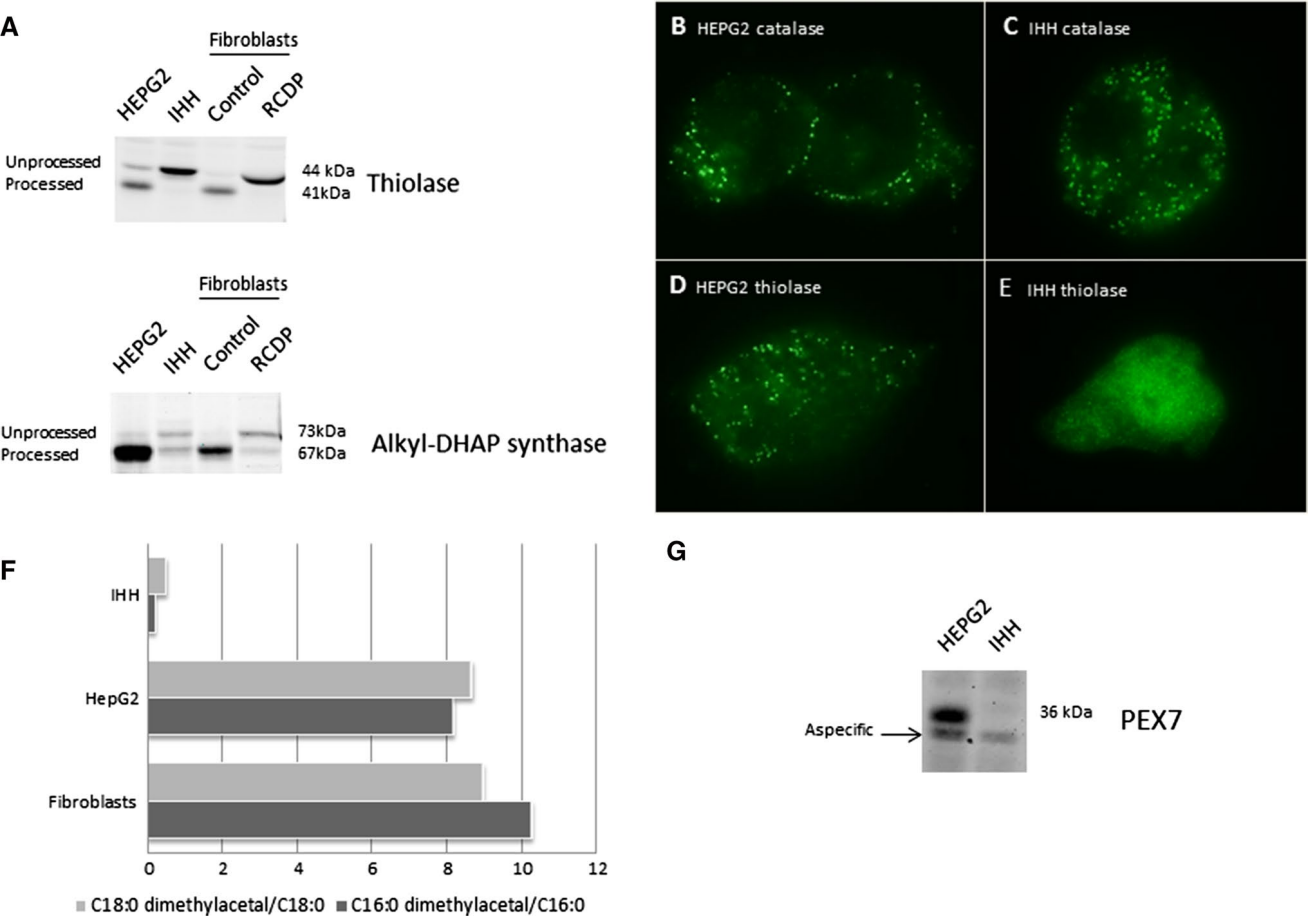
**a** Immunoblot analysis using antibodies against peroxisomal 3-ketoacyl-CoA thiolase and alkyl-DHAP synthase. **b** Immunofluorescence microscopy analysis using antibodies against catalase in HepG2 and IHH (**c**) shows a peroxisomal fluorescence signal. Immunofluorescence microscopy analysis using antibodies against peroxisomal 3-ketoacyl-CoA thiolase shows a peroxisomal fluorescence signal in HepG2 (**d**), but a cytosolic signal in IHH (**e**). **f** Relative amount of plasmalogens expressed as the ratio of C16:0 dimethylacetal and C18:0 dimethylacetal to their corresponding fatty acid methyl ester. **g** Immunoblot analysis using an antibody against the c terminus of PEX7

In order to characterize the peroxisomal functions of the IHH cell line, we measured β-oxidation activities using pristanic acid or cerotic acid (C26:0) as substrates, and phytanic acid α-oxidation activity, and compared these with the activities in HepG2 cells and control primary skin fibroblasts. The β-oxidation rates of pristanic acid and cerotic acid (C26:0) were similar or higher in IHH cells when compared to those in HepG2 cells and control fibroblasts, respectively (Fig. 1a). In contrast, however, the α-oxidation of phytanic acid was markedly impaired in IHH cells (Fig. 1b). Impaired phytanic acid α-oxidation in conjunction with normal β-oxidation can be due to an isolated defect of phytanoyl-CoA hydroxylase, as in adult Refsum’s disease (Jansen et al. 1998), or to a defect of the import of this PTS2-targeted peroxisomal enzyme, as in RCDP type 1 (Braverman et al. 1997). We further evaluated PTS2-mediated protein import in the IHH cells by immunoblot analysis using antibodies against the PTS2-targeted peroxisomal proteins 3-ketoacyl-CoA thiolase and alkyl-DHAP synthase. We only detected the unprocessed precursors of these proteins in homogenates of the IHH cells, indicating that they were not imported into peroxisomes where processing into the corresponding mature proteins usually occurs. The same unprocessed precursors are observed in homogenates of fibroblasts from a PEX7-deficient RCDP type 1 patient (Fig. 2a).

The specific defect in peroxisomal import of PTS2-targeted proteins was confirmed by immunofluorescence microscopy of IHH cells, using antibodies against 3-ketoacyl-CoA thiolase and PTS1-targeted catalase. Antibodies against catalase showed a punctate peroxisomal fluorescence pattern in IHH cells and HepG2 cells, indicating that PTS1-targeted proteins are normally imported. In contrast, a cytosolic fluorescence signal was seen in IHH cells when using antibodies against 3-ketoacyl-CoA thiolase, whereas HepG2 cells showed a punctate peroxisomal fluorescence pattern (Fig. 2b–e).

The PTS2-targeted peroxisomal enzyme alkyl-DHAP synthase is known to play a crucial role in the biosynthesis of plasmalogens (ether lipids) (Wanders and Waterham 2006). Accordingly, when we measured plasmalogen levels in the IHH cells, HepG2 cells and control fibroblasts a marked plasmalogen deficiency in IHH cells was found (Fig. 2f).

Since our combined data clearly pointed to a defect of PEX7, we sequenced the coding region of *PEX7* in the IHH cells. No mutations were found in exons 2–10, but we were unable to PCR-amplify exon 1, suggesting a deletion including exon 1 of this gene, which thus prevents the synthesis of PEX7 protein. In addition, no PEX7 protein could be detected in homogenates of IHH cells by immunoblot analysis using an antibody against the c terminus of PEX7 (Fig. 2g). The defect in PEX7 was confirmed by restoration of peroxisomal PTS2-mediated protein import in IHH cells after transfection with control PEX7 cDNA (not shown). Since it was recently reported that mutations in the PEX5L-specific exon 9 of *PEX5* can also cause deficient import of PTS2-targeted proteins only (Barøy et al. 2015), we also performed genetic complementation of IHH cells with PEX5L cDNA. Restoration of PTS2-mediated protein import did not occur (not shown).

Our findings show that the IHH cell line has a defect in PEX7, which results in a marked plasmalogen deficiency and impairment of phytanic acid α-oxidation and thus affects lipid homeostasis in these cells. This defect must have been introduced during the immortalization procedure, because the individual whose liver biopsy was used to generate the cell line did not clinically present with RCDP (Schippers et al. 1997). These findings may have important implications both for interpretation of data previously generated using this cell line and when considering using this cell line for future research. The extent to which these abnormalities impact processes important in liver metabolism remains to be determined. However, multiple studies have suggested a role for plasmalogens in cholesterol trafficking (Braverman and Moser 2012) and it was recently reported that low levels of plasmalogens influence cholesterol biosynthesis (Honsho et al. 2015). The IHH cell line is a good model to study the effect of plasmalogen deficiency on different metabolic pathways.

## Abbreviations

IHH: Immortalized human hepatocyte
SV40 Tag: Simian virus 40 large T antigen
PTS: Peroxisomal targeting signal
DHAP: Dihydroxyacetone phosphate
RCDP: Rhizomelic chondrodysplasia punctata
